# Osteolytic metatarsal osteomyelitis regenerated by combined treatment of artificial carbon dioxide foot bathing and povidone–iodine sugar ointment: a case report

**DOI:** 10.1186/s13256-022-03654-7

**Published:** 2022-11-21

**Authors:** Masakatsu Hihara, Michika Fukui, Toshihito Mitsui, Natsuko Kakudo, Atsuyuki Kuro

**Affiliations:** Department of Plastic and Reconstructive Surgery, Kansai Medical University, 2-5-1, Shin-Machi, Hirakata, Osaka 573-1010 Japan

**Keywords:** Artificial carbon dioxide foot bathing, Povidone–iodine sugar ointment, Diabetic foot ulcers, Bone and joint regeneration, Osteomyelitis, Healthcare costs

## Abstract

**Background:**

In recent years, the number of patients with ischemic skin ulcers due to diabetes mellitus and arteriosclerosis obliterans are increasing. Accordingly, endovascular therapy, drugs, and various wound dressings have been developed and applied to diabetic foot ulcers, and negative-pressure wound therapy, which often requires expensive and burdensome procedures for medical personnel, has also become popular. So simple and minimal invasive home treatment by the patient or their caregiver is required.

**Case presentation:**

The present patient (77 years old, male, Asian) had developed left sole ulcers with draining pus that were resistant to conventional treatment, and he suffered from gait disturbance. We report a case of metatarsal osteomyelitis in a patient with diabetes mellitus and arteriosclerosis obliterans, in whom artificial carbon dioxide foot bathing and povidone–iodine sugar ointment were used continuously to promote bone and joint regeneration, and skin ulcer healing.

**Conclusions:**

A simple therapeutic intervention with artificial carbon dioxide foot bathing and povidone–iodine sugar ointment can improve not only ischemic skin ulcers, but also the bone and joint regeneration of ischemic limbs. This therapy can lead to a reduction in healthcare costs for a huge number of diabetic patients.

## Introduction

In recent years, the number of patients with ischemic skin ulcers due to diabetes mellitus and arteriosclerosis obliterans has been increasing. Accordingly, endovascular therapy, growth factors (prostaglandins, trafermin, and so on) and various wound dressings have been developed and applied to diabetic foot ulcers, and negative-pressure wound therapy (NPWT), which often requires expensive and burdensome procedures for medical personnel, has also become popular [1].

In cases of complicated osteomyelitis, current standard treatments begin with surgical debridement, including sequestrectomy, but mechanical and uniform sequestrectomy may constitute “over-surgery.” Artificial carbon dioxide bathing has been reported as a therapeutic intervention to improve the cutaneous circulation of ischemic limbs and ischemic skin ulcers.

In this study, we report a case of metatarsal osteomyelitis in a patient with diabetes mellitus and arteriosclerosis obliterans, in whom artificial carbon dioxide foot bathing and povidone–iodine sugar ointment were used continuously to promote bone and joint regeneration, and skin ulcer healing.

## Case presentation

**Fig. 1:**
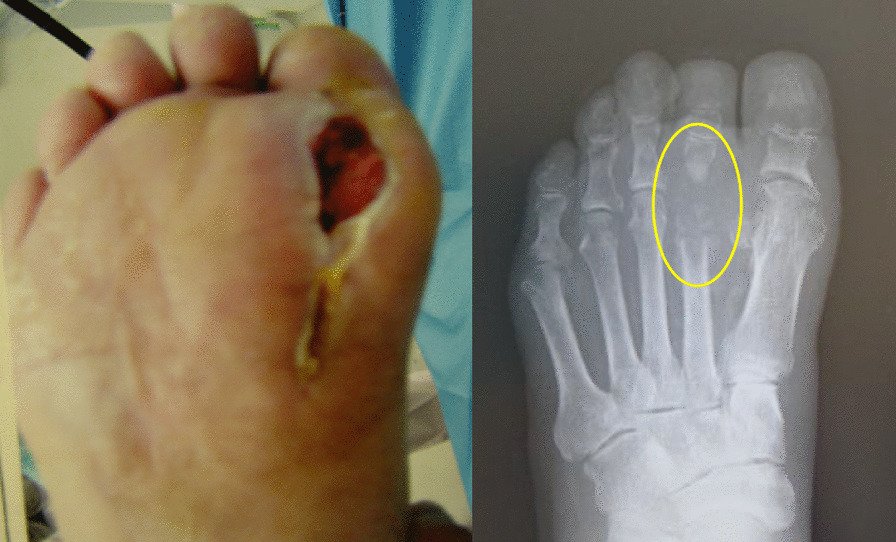
Pus discharge of the right plantar ulcer increased and the metatarsophalangeal joint of the second toe was destroyed and showed osteolytic changes. Yellow circle indicates osteolytic areas

A 77-year-old Asian man with diabetes mellitus, arteriosclerosis obliterans, and hypertension developed persistent plantar ulcers after percutaneous transluminal angioplasty (PTA) of the right lower extremity, which was performed by his previous physician. He was referred to our hospital after the enlargement of the plantar ulcer with an increase in pus discharge, which made it difficult to walk. Radiography revealed that the metatarsophalangeal joint of the second toe was destroyed and showed osteolytic changes (Fig. 1). A biochemical analysis revealed the following: white blood cell (WBC), 9.710^9^/L; red blood cell (RBC), 4.210^12^/L; c-reactive protein (CRP), 13.8 mg/L; and hemoglobin (Hb)A1c, 6.8% (National Glycohemoglobin Standardization Program; NGSP). Methicillin-resistant *Staphylococcus aureus* (MRSA) was detected in the wound. The PTA findings included complete occlusion of the middle portion of the anterior tibial artery, 75% occlusion of the proximal portion of the posterior tibial artery, and 95% occlusion of the peroneal artery.

**Fig. 2:**
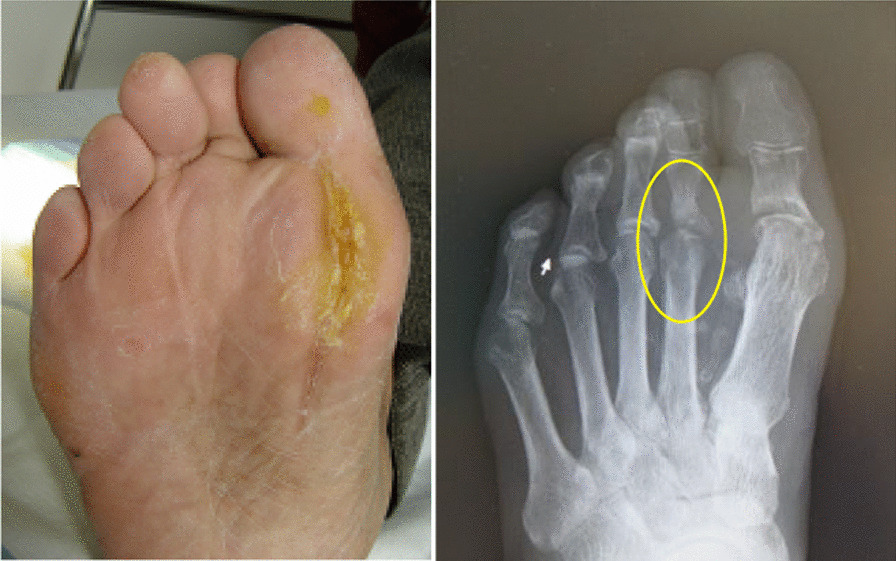
One year and five months later, the wound was healing and radiographs showed the regeneration of a joint-like structure in the metatarsophalangeal joint of the second toe. Yellow circle indicates the regeneration of a joint-like structure

Because no fever was observed and an immediate incision could be performed, systemic antibiotics were not administered. An extended plantar incision was performed at the pus drainage hole between the first and second metatarsophalangeal (MTP) joint to a depth just above the periosteum under local anesthesia. No sequestrectomy or removal of flexor tendons was performed, as is commonly done. After making an extended incision, the patient was instructed to take a daily foot bath in carbonated water (AS care; Asahi Kasei Medical Co., Ltd., Tokyo, Japan) at 37 °C with a carbon dioxide concentration of 1000–1300 ppm for 15 minutes at home. We also advised him to continue applying povidone–iodine sugar ointment (U-PASTA; Kowa Company, Ltd., Nagoya, Japan) to the wound after the foot bath. After 6 months of biweekly wound examinations, wound closure and bone and joint remodeling were observed, and the therapy was concluded. At 1 year 5 months later, the lesion had not recurred, and radiography demonstrated the regeneration of a joint-like structure in the metatarsophalangeal joint of the second toe, allowing the patient to walk normally (Fig. 2): an unexpected event in the course of treating this patient. Since this method uses carbonic acid and sugar, we tentatively called it soda pop therapy.

## Discussion

The effects of carbonic acid springs have long been known to include flushing of the skin at the site of immersion, increased blood flow in the skin and muscle, decreased blood pressure, and bradycardia [2]. The efficacy of high-concentration carbon dioxide foot bathing on ischemic limbs and skin ulcers in the clinical setting has already been reported [3, 4]. In Japan, tablets for generating artificial carbonated water are commercially available (AS care; Asahi Kasei Medical Co., Ltd.) and are frequently added to foot baths to improve blood flow in the legs of dialysis patients. AS care contains carbonate, sodium dichloroisocyanurate, and sodium dichloroisocyanurate hydrolyzes to produce hypochlorous acid (HOCl) simultaneously with carbon dioxide generation. Transdermally absorbed carbon dioxide is converted to bicarbonate ions, which act directly on endothelial cells to increase nitric oxide (NO) production through phosphorylation of endothelial nitric oxide synthase (eNOS), a process that is considered to improve blood flow [5]. Hypochlorous acid (HOCl) has both antimicrobial and deodorizing effects on wounds [6]. The optimal conditions for improving skin blood flow are as follows: carbon dioxide gas concentration, 1000–1300 ppm; water temperature, 37 °C; bathing time, 15 minutes; and application interval, once a day [7]. However, the osteogenic effect that suggests the improvement of bone metabolism has only been reported in animal experiments [8, 9].

It has been suggested that povidone–iodine sugar ointment (U-PASTA; Kowa Company, Ltd. Japan), which is widely used for incurable ulcers, activates keratinocytes and fibroblasts and promotes wound healing [10], and its effectiveness in improving wound healing has been proven in clinical surgical wounds [11]. Povidone–iodine sugar ointment, which consists of 70% sugar and 3% povidone–iodine, also called Knutson’s formula, has been proposed to improve wound healing due to a reduction in bacterial contamination, rapid debridement of eschar, probable nourishment of surface cells, filling of defects with granulation tissue, and covering of granulation tissue with epithelium [12].

In this study, we applied the combination of these treatments in a case of metatarsal osteomyelitis caused by diabetic foot ulcer, and were able to regenerate the destroyed metatarsophalangeal joint of the second toe, which showed osteolytic changes. There have been no clinical reports on the stimulation of bone and joint regeneration by carbon dioxide foot bathing and povidone–iodine sugar ointment. The synergistic effects of carbon dioxide foot bathing on increased muscle blood flow and the epithelializing interaction of povidone–iodine sugar ointment may be the mechanism underlying this breakthrough result. It is speculated that bone cortex may have regenerated around the remaining bone marrow in areas that were difficult to visualize on X-ray. However, there is no evidence that this is the case. This result also suggests that aggressive debridement or curettage of the lesion may not always be necessary, even if osteolytic changes are seen in osteomyelitis.

As the number of patients for whom surgical intervention is difficult (for example patients on dialysis) is increasing, this therapy is considered to be extremely useful because of its minimal invasiveness and low cost, which allows simple home treatment by the patient or their caregiver. There have been no previous clinical reports on bone and joint regeneration stimulation by artificial carbon dioxide foot bathing and povidone–iodine sugar ointment. This therapy may lead to a reduction in healthcare costs for a huge number of diabetic patients.

## Conclusion

Although it has been reported that carbon dioxide foot bathing can improve cutaneous circulation in patients with conditions such as ischemic ulcers, no clinical studies have reported the bone metabolism dynamics. We reported a case of metatarsal osteomyelitis associated with diabetes mellitus and arteriosclerosis obliterans in which bone and joint regeneration was promoted by the application of artificial carbon dioxide foot bathing and povidone–iodine sugar ointment.

## Data Availability

Not applicable.
